# Impact of Pulse Pressure on Acute Brain Injury in Venoarterial ECMO Patients with Cardiogenic Shock During the First 24 Hours of ECMO Cannulation: Analysis of the Extracorporeal Life Support Organization Registry

**DOI:** 10.21203/rs.3.rs-3646443/v1

**Published:** 2023-11-23

**Authors:** Andrew Kalra, Jin Kook Kang, Christopher Wilcox, Patricia Brown, Peter Rycus, Marc M Anders, Akram M Zaaqoq, Daniel Brodie, Glenn J R Whitman, Sung-Min Cho

**Affiliations:** Johns Hopkins University School of Medicine; Johns Hopkins University School of Medicine; Mercy Hospital of Buffalo; Johns Hopkins University School of Medicine; Extracorporeal Life Support Organization; Baylor College of Medicine; University of Virginia; Johns Hopkins University School of Medicine; Johns Hopkins University School of Medicine; Johns Hopkins University School of Medicine

**Keywords:** venoarterial extracorporeal membrane oxygenation, pulse pressure, acute brain injury, Extracorporeal Life Support Organization Registry, ischemic and hemorrhagic stroke

## Abstract

**Background:**

Low pulse pressure (PP) in venoarterial-extracorporeal membrane oxygenation (VA-ECMO) is a marker of cardiac dysfunction and has been associated with acute brain injury (ABI) as continuous-flow centrifugal pump may lead to endothelial dysregulation.

**Methods:**

We retrospectively analyzed adults (≥18 years) on “peripheral” VA-ECMO support for cardiogenic shock in the Extracorporeal Life Support Organization Registry (1/2018–7/2023). Cubic splines were used to establish a threshold (PP≤10 mmHg at 24 hours of ECMO support) for “early low” PP. ABI included central nervous system (CNS) ischemia, intracranial hemorrhage, brain death, and seizures. Multivariable logistic regressions were performed to examine whether PP≤10 mmHg was associated with ABI. Covariates included age, sex, body mass index, pre-ECMO variables (temporary mechanical support, vasopressors, cardiac arrest), on-ECMO variables (pH, PaO_2_, PaCO_2_), and on-ECMO complications (hemolysis, arrhythmia, renal replacement therapy).

**Results:**

Of 9,807 peripheral VA-ECMO patients (median age=57.4 years, 67% male), 8,294 (85%) had PP>10 mmHg vs. 1,513 (15%) had PP≤10 mmHg. Patients with PP≤10 mmHg experienced ABI more frequently vs. PP>10 mmHg (15% vs. 11%, p<0.001). After adjustment, PP≤10 mmHg was independently associated with ABI (adjusted odds ratio [aOR]=1.25, 95% confidence interval [CI]=1.06–1.48, p=0.01). CNS ischemia and brain death were more common in patients with PP≤10 mmHg vs. PP>10 mmHg (8% vs. 6%, p=0.008; 3% vs. 1%, p<0.001). PP≤10 mmHg was associated with CNS ischemia (aOR=1.26, 95%CI=1.02–1.56, p=0.03) but not intracranial hemorrhage (aOR=1.14, 95%CI=0.85–1.54, p=0.38).

**Conclusions:**

Early low PP (≤10 mmHg) at 24 hours of ECMO support was associated with ABI, particularly CNS ischemia, in peripheral VA-ECMO patients.

## Introduction

Venoarterial extracorporeal membrane oxygenation (VA-ECMO) is increasingly used to treat patients with refractory cardiogenic shock (CS).^1–4^ Acute brain injury (ABI), including intracranial hemorrhage (ICH), ischemic stroke, and hypoxic-ischemic brain injury (HIBI) occurs in up to 20% of adults on VA-ECMO support and is associated with increased mortality risk.^5^ Blood pressure variables, such as pulse pressure (PP), defined as the difference between systolic (SBP) and diastolic (DBP) blood pressure, have been shown to be important surrogate markers of cardiovascular function in patients on mechanical circulatory support.^6 7, 8^

VA-ECMO operates with a continuous-flow centrifugal pump, which is associated with endothelial dysregulation/dysfunction^9, 10^ Although the precise mechanism is not entirely understood, this ensuing endothelial cell dysregulation resulting from nonpulsatile flow predisposes patients to neurological injury such as ABI. Therefore, PP may be a good surrogate marker for predicting neurological outcomes in ECMO patients. A recent study demonstrated PP < 20 mmHg within 12 hours of ECMO cannulation was associated with ABI in 123 VA-ECMO patients.^11^ Still, this study was limited by small sample size, from single center, and including central VA-ECMO and post-cardiotomy shock patients who are at higher predisposition of ABI.^12^ Furthermore, peripheral VA-ECMO patients have hemodynamics states that are associated with vascular and perfusion abnormalities^13^ and are thus an important population to investigate the association between PP and ABI.

Using the largest registry of ECMO patients globally, the Extracorporeal Life Support Organization (ELSO) Registry, we sought to investigate the association between early PP and ABI in peripheral VA-ECMO patients. We hypothesized that low PP in the first 24 hours of ECMO support was independently associated with higher occurrence of ABI.

## Methods

### Study design and population

This study was approved by the Johns Hopkins Hospital Institutional Review Board with a waiver of informed consent since this was a retrospective observational study (IRB00216321). The ELSO Registry is an international multicenter registry from over 500 ECMO centers.^14^ The Registry collects demographics, pre-ECMO comorbidities, pre-ECMO and on-ECMO hemodynamic and arterial blood gas (ABG) information, on-ECMO neurological and other systemic complications, and outcomes such as mortality.^15^ Comorbidity information was recorded using the *International Classification of Diseases, 10th Revision (ICD-10)* codes.

We included patients who were 1) 18 years of age or older; and 2) supported with “peripheral” VA-ECMO and diagnosed with CS from 2018–2023. We excluded repeat ECMO runs within the same patient to avoid complexity and bias. We also excluded patients with missing blood pressure (systolic and diastolic at 24 hours of ECMO support) and cannulation information, central cannulation, on-ECMO percutaneous ventricular assist device or central venous access device support, coronary artery bypass graft or percutaneous coronary intervention, and post-cardiotomy shock. Patients with these conditions were excluded as they could impact the interpretation of PP readings and their association with ABI.

### Data collection

The ELSO Registry collects ABG and hemodynamic information before and after ECMO cannulation (i.e., “pre-ECMO” and “on-ECMO”, respectively). Pre-ECMO ABGs were drawn at maximum 6 hours before ECMO cannulation, and pre-ECMO ventilator settings were recorded within 6 hours of ECMO cannulation. If multiple ABGs existed within a specific duration, the pre-ECMO ABG that was closest to the beginning of ECMO cannulation was selected. On-ECMO ABGs were drawn after ECMO cannulation started, no longer than 30 hours post-cannulation. If multiple ABGs were taken, the on-ECMO ABG nearest to 24 hours after the start of cannulation was chosen. On-ECMO hemodynamics were gathered closest to 24 hours after ECMO cannulation, though they could be collected at 18–30 hours after cannulation. Each variable was abstracted by a trained ELSO data manager/abstracter and was collected simultaneously.

### Definitions

On-ECMO PP was calculated as “SBP at 24 hours” - “DBP at 24 hours”. Delta partial pressure of arterial carbon dioxide (PaCO_2_) was calculated as “On-ECMO PaCO_2_ at 24 hours” - “Pre-ECMO PaCO_2_”. Pre-ECMO ventilator settings included conventional ventilation, high-frequency oscillatory ventilation, other high frequency ventilation (high frequency jet ventilation or percussive ventilation), other non-specified ventilations, and absence of ventilation. Pre-ECMO additional temporary mechanical circulatory support (tMCS) included intra-aortic balloon pump (IABP), Impella^®^, and left and right ventricular assist devices (though patients supported with on-ECMO tMCS were excluded from the analysis, as previously described). Pre-ECMO vasopressor infusions included dopamine, epinephrine, norepinephrine, phenylephrine, and vasopressin. Infusions were treated as a binary variable, meaning we treated them as the presence or absence of the infusions. Pre-ECMO vasopressor infusions were utilized for at least 6 hours within 24 hours of the start of ECMO cannulation. Pre-ECMO cardiac arrest was defined as an event that required the use of cardiopulmonary resuscitation in conjunction with the administration of external cardiac massage within 24 hours of ECMO cannulation. Central cannulation was defined as placement of the reinfusion cannula directly into the aorta. Peripheral cannulation was defined as placement of cannula in a site other than the aorta (peripheral vessels).

On-ECMO complications included cardiac arrhythmia, hemolysis, renal replacement therapy, gastrointestinal hemorrhage, and ECMO circuit failure. Definitions for each complication are in the **Supplemental Methods.**

ABI was defined as the presence of central nervous system (CNS) infarction (ischemic stroke), diffuse ischemia (hypoxic-ischemic brain injury, HIBI), intra/extra parenchymal hemorrhage, intraventricular hemorrhage, seizures determined by electroencephalograph or clinically, and neurosurgical intervention (examples include intracranial pressure monitor, external ventricular drain, and craniotomy) and brain death during ECMO support. CNS ischemia was defined as ischemic stroke (determined by ultrasound, computed tomography, CT, or magnetic resonance imaging, MRI) and HIBI (determined by CT or MRI). ICH included intra/extra parenchymal hemorrhage and intraventricular hemorrhage (both determined by CT or MRI).

### Outcomes

The primary outcome was ABI during ECMO support between patients with PP ≤ 10 vs. PP > 10 mm Hg. The secondary outcomes were subtypes of ABI, CNS ischemia and ICH.

### Statistical Analysis

Continuous variables were represented as median with interquartile range (IQR). Categorical variables were presented as frequency with percentages. The Wilcoxon rank-sum and Pearson’s chi-square tests were utilized to compare continuous and categorical variables, respectively. Differences in PP between those with ABI vs. those without ABI were compared with Wilcoxon rank-sum. Statistical significance was set at a p-value < 0.05. Data missingness was handled with multiple imputation with five separately imputed datasets (Rubin’s Rules)^16^ to augment statistical power. Continuous, unordered categorical, and dichotomous missing variables were imputed using regression with predictive mean matching, polytomous logistic regression, and logistic regression. All missing variables are shown in **Supplemental Table 1.**

Cubic spline analysis was utilized to non-linearly model the impact of PP on ABI. Based on inflection points (“spline knots”) in this model, combined with prior data and clinical knowledge, we determined an appropriate PP threshold (≤ 10 mmHg) for logistic regression analysis. Boxplots were used to descriptively portray the association between PP vs. ABI. We performed univariable and multivariable logistic regression for ABI, CNS ischemia, and ICH in peripherally cannulated patients to determine if PP ≤ 10 mmHg was a significant risk factor for each of these outcomes even after adjustment for clinically relevant covariates. We chose covariates selected *a priori* based on clinical judgement and prior data for each model.^5, 17^ Adjusted covariates in the ABI model included age, sex, body mass index, pre-ECMO variables (additional tMCS, vasopressor infusions, cardiac arrest), on-ECMO variables (pH, arterial partial pressure of oxygen, PaO_2_), delta PaCO_2_, and on-ECMO complications (hemolysis, arrhythmia, renal replacement therapy). In the CNS ischemia (ischemic stroke or HIBI) model, age, sex, pre-ECMO variables (PaCO_2_, PaO_2_, pH, vasopressor infusions, cardiac arrest), delta PaCO_2_, and on-ECMO complications (ECMO circuit failure, arrhythmia) were included in the adjustment. In the ICH model, age, sex, pre-ECMO variables (additional tMCS, vasopressor infusions, cardiac arrest), on-ECMO variables (PaO_2_, pH), delta PaCO_2_, and on-ECMO complications (ECMO circuit failure, gastrointestinal hemorrhage, hemolysis, renal replacement therapy) were included in the adjustment. In an exploratory analysis, we performed a multivariable logistic regression model for central VA-ECMO patients to determine if PP was associated with ABI. Adjusted odds ratios (aOR) were presented with 95% confidence intervals (CIs). All statistical analyses were performed using R Studio (R 4.1.2, www.r-project.org).

## Results

### Study population

**Figure 1:**
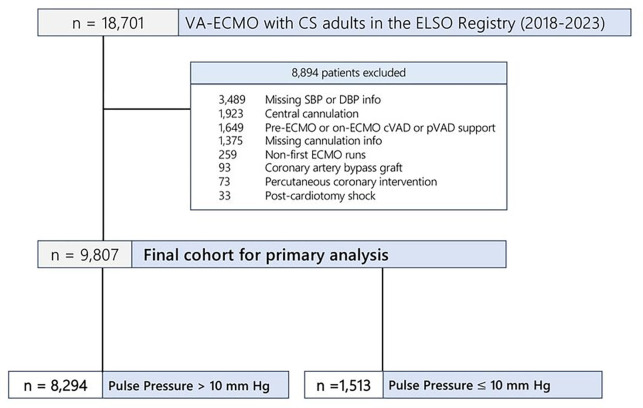
Flow diagram for the creation of our study cohort.

Of 18,701 VA-ECMO patients with CS, we included 9,807 patients in our study after the inclusion and exclusion criteria (Fig. 1). Cubic spline analysis of the ELSO Registry showed an inflection point at a PP of 10 mmHg when non-linearly modeling the impact of pulse pressure on ABI; therefore, we chose this value as our threshold in our analysis. Of 9,807 peripheral VA-ECMO patients (median age = 57.4 years, 67% male), 8,294 (85%) had a PP > 10 mmHg vs. 1,513 (15%) had a PP ≤ 10 mmHg (Table 1). Age, sex, body mass index, and race/ethnicity were similar between both groups. The median duration of ECMO support was 4.9 days (IQR = 2.9–8.0).

A total of 1,096 (11.1%) patients experienced ABI. Patients with PP ≤ 10 mmHg experienced ABI more frequently than those with PP > 10 mmHg (n = 220, 15% vs. n = 876, 11%, p < 0.001, **Supplemental Table 2**). Overall, 608 (6.2%) patients experienced CNS ischemia (ischemic stroke or HIBI) and 311 (3.2%) patients experienced ICH. CNS ischemia and brain death were more common in patients with PP ≤ 10 mmHg vs. those with PP > 10 mmHg (n = 121, 8% vs. n = 487, 6%, p = 0.002; n = 43, 3% vs. n = 124, 1%, p < 0.001). Patients with PP ≤ 10 mmHg were more likely to die vs. those with PP > 10 mmHg (n = 1,021, 67% vs. n = 3,813, 46%, p < 0.001).

### Acute brain injury

**Figure 2:**
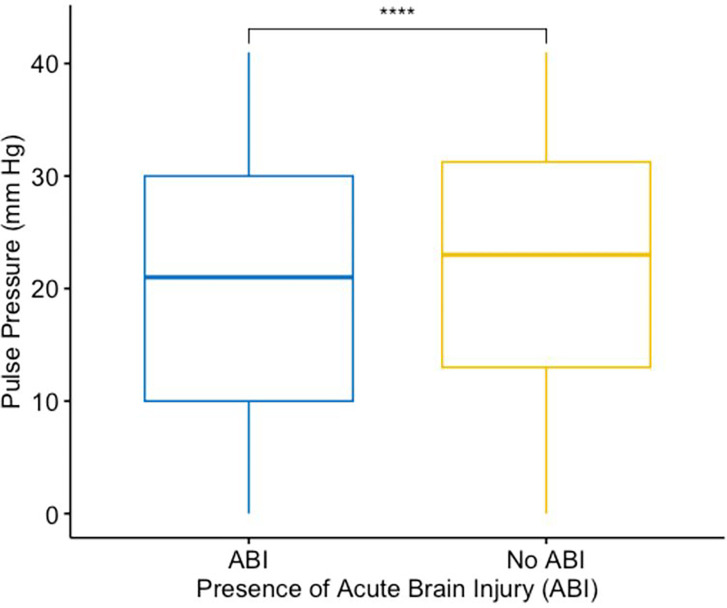
Boxplot of pulse pressure (y-axis) vs. peripheral venoarterial extracorporeal membrane oxygenation patients with acute brain injury and those without acute brain injury (x-axis).

Baseline clinical characteristics and demographics were also compared between patients with ABI vs. without ABI (**Supplemental Table 2**). Patients with ABI had a lower on-ECMO PP vs. those without ABI [median (IQR), 27(14–42) vs. 30(17–44) mmHg, p < 0.001] (Fig. 2)

**Figure 3:**
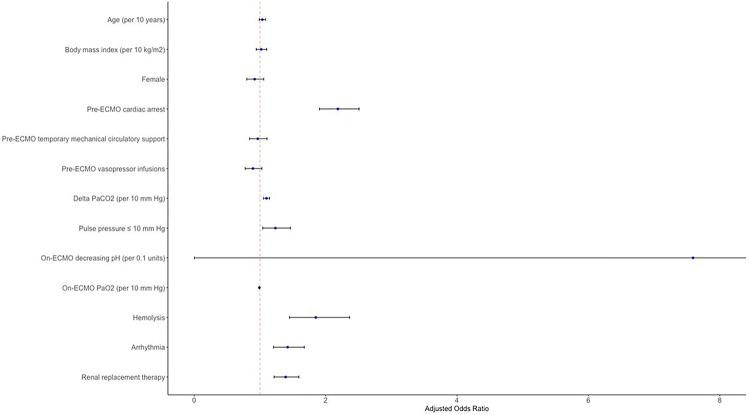
Forest plot of multivariable logistic regression model for occurrence of acute brain injury in peripheral venoarterial extracorporeal membrane oxygenation patients.

In a multivariable logistic regression after adjusting for pre-selected clinically relevant covariates, on-ECMO PP ≤ 10 mmHg was independently associated with ABI (aOR = 1.25, 95%CI = 1.06–1.48, p = 0.01, Table 2, Fig. 3). Additional risk factors associated with ABI included a higher delta PaCO_2_ (aOR = 1.10 per 10 mmHg increase, 95%CI = 1.05–1.15, p < 0.001), pre-ECMO cardiac arrest (aOR = 2.05, 95%CI = 1.78–2.36, p < 0.001), hemolysis (aOR = 1.81, 95%CI = 1.42–2.32, p < 0.001), arrhythmia (aOR = 1.41, 95%CI = 1.19–1.66, p < 0.001), and renal replacement therapy (aOR = 1.41, 95%CI = 1.23–1.61, p < 0.001). Higher on-ECMO PaO_2_ (aOR = 0.99 per 10 mmHg increase, 95%CI = 0.986–0.996, p < 0.001) was protective against ABI.

### Central nervous system ischemia (ischemic stroke and hypoxic-ischemic brain injury)

**Figure 4:**
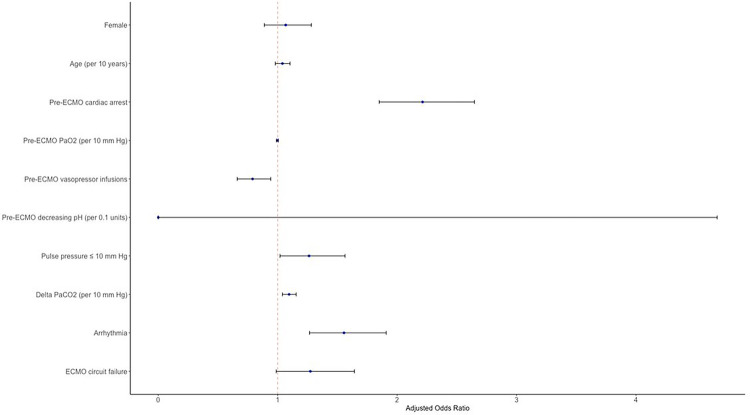
Forest plot of multivariable logistic regression model for occurrence of central nervous system ischemia in peripheral venoarterial extracorporeal membrane oxygenation patients.

In a multivariable logistic regression, on-ECMO PP ≤ 10 mmHg was independently associated with CNS ischemia (aOR = 1.26, 95%CI = 1.02–1.56, p = 0.03, **Supplemental Table 3**, Fig. 4). On-ECMO PP ≤ 10 mmHg was also independently associated with ischemic stroke by itself (aOR = 1.34, 95%CI = 1.04–1.72, p = 0.02). Additional risk factors associated with CNS ischemia included delta PaCO_2_ (aOR = 1.10 per 10 mmHg increase, 95%CI = 1.04–1.15, p < 0.001), arrhythmia (aOR = 1.55, 95%CI = 1.27–1.91, p < 0.001), and pre-ECMO cardiac arrest (aOR = 2.21, 95%CI = 1.85–2.65, p < 0.001). Pre-ECMO vasopressor infusions (aOR = 0.79, 95%CI = 0.66–0.94, p = 0.01) were protective against CNS ischemia.

### Intracranial hemorrhage

**Figure 5:**
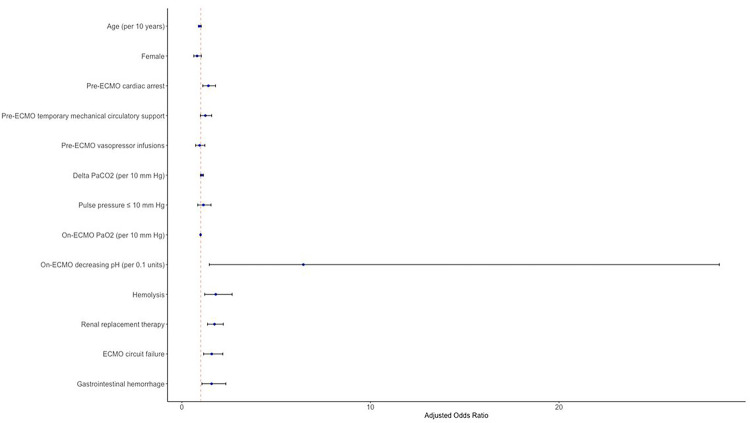
Forest plot of multivariable logistic regression model for occurrence of intracranial hemorrhage in peripheral venoarterial extracorporeal membrane oxygenation patients

In a multivariable logistic regression, on-ECMO PP ≤ 10 mmHg was not significantly associated with ICH (aOR = 1.14, 95%CI = 0.85–1.54, p = 0.38, **Supplemental Table 4**, Fig. 5). Risk factors associated with ICH included hemolysis (aOR = 1.80, 95%CI = 1.21–2.67, p < 0.001), renal replacement therapy (aOR = 1.73, 95%CI = 1.36–2.20, p < 0.001), ECMO circuit failure (aOR = 1.58, 95%CI = 1.15–2.17, p < 0.001), gastrointestinal hemorrhage (aOR = 1.58, 95%CI = 1.07–2.33, p = 0.02), and pre-ECMO cardiac arrest (aOR = 1.41, 95%CI = 1.11–1.78, p = 0.01).

### Exploratory analysis – central cannulation

In a univariable logistic regression analysis for ABI, on-ECMO PP ≤ 10 mmHg was also significantly associated with ABI in central VA-ECMO patients (OR = 1.40, 95%CI = 1.01–1.93, p = 0.04) and for ischemia (OR = 1.56, 95%CI = 1.08–2.25, p = 0.02). In a multivariable logistic regression, on-ECMO PP ≤ 10 mmHg was not associated with ABI (aOR = 1.17, 95%CI = 0.83–1.64, p = 0.38), CNS ischemia (aOR = 1.46, 95%CI = 0.99–2.13, p = 0.05,), or ICH (aOR = 1.35, 95%CI = 0.71–2.56, p = 0.36), in central VA-ECMO patients (**Supplemental Tables 5–7).**

## Discussion

In this ELSO Registry analysis of 9,807 peripheral VA-ECMO patients, we found that a low PP (≤ 10 mmHg) measured at 24-hours of ECMO support was independently associated with greater occurrence of ABI in peripheral VA-ECMO patients after adjusting for pre-selected clinically relevant covariates. Additionally, low PP was associated with CNS ischemia but not ICH. We also identified other risk factors for ABI in peripheral VA-ECMO patients: higher delta PaCO_2_, pre-ECMO cardiac arrest, and on-ECMO hemolysis, cardiac arrhythmia, and renal replacement therapy.

There may be multiple mechanisms at play that can lead to PP influencing ABI in this cohort. One potential mechanism relates to the loss of pulsatility that occurs inherently with VA-ECMO and is enhanced by adjustment of pump speeds,^18–20^ as nonpulsatile flow is associated with elevated vascular resistance, increased muscular sympathetic nervous system activity, coronary artery disease, limited oxygen consumption, and interruption of cerebral autoregulation.^21–23^ These factors have been associated with increased incidence of ABI^24–29^ in non-ECMO patients. Additionally, patients undergoing coronary artery bypass graft with IABP and cardiopulmonary bypass (i.e., nonpulsatile flow) have been shown to have less endothelial activation^30–33^ and to observe a reduction in nitric oxide^34, 35^ due to systemic inflammatory response syndrome.^9, 36, 37^ Patients on the ECMO circuit frequently experience extreme changes in hemodynamic parameters such as PaO_2_^17^ and PaCO_2_,^38–40^ which were previously shown to be associated with ABI. These blood gas derangements, combined with already compromised endothelium function and nonpulsatile cerebral blood flow,^41^ may lead to ABI. Additionally, as left ventricular (LV) venting may lower PP and further predispose VA-ECMO patients to ABI, future research is warranted to investigate the effects of tMCS such as IABP and Impella^®^ on the association between PP and ABI.

Our results demonstrated that low PP was associated with CNS ischemia but not ICH in peripheral VA-ECMO. Induced by ECMO circuit, the absence of pulsatility is associated with reduced O_2_ consumption and impaired cerebral autoregulation, potentially contributing to CNS ischemia.^21^ Furthermore, low PP can indicate inadequate cardiac contractility, and thus systemic hypoperfusion, which may increase the risk of CNS ischemia. Interestingly, in contrast to previous literature describing that a larger delta PaCO_2_ was associated with ICH,^42^ our study demonstrated that delta PaCO_2_ was associated with CNS ischemia rather than ICH. These results did not persist in central VA-ECMO which may be due to different hemodynamic states between both cohorts.^13^ Other additional key factors such as the use of systemic anticoagulation, duration of ECMO support, hemolysis, and platelet imbalance may also be involved in the pathophysiology of ICH.^43^ Overall, these findings suggest that additional research is necessary to clarify how certain risk factors lead to either CNS ischemia or ICH in peripheral VA-ECMO patients.

Interestingly, unlike a previous study suggesting severe hyperoxia is associated with ABI,^17^ an increase in PaO_2_ was a protective factor for ABI in our analysis. One explanation is that aggressive oxygen therapy helps mitigate the effects of potential hypoxemia during ECMO support as hypoxemia in ECMO patients is well documented.^44, 45^ Furthermore, a higher PaO_2_ may help increase regional brain oxygen tension^46^ and accordingly improve the cerebral metabolic rate of oxygen. We also note we did not “bin” PaO_2_ values (continuous variable) by groups as this previous study did which may increase their risk of bias.^47^ Overall, these results suggest that additional research with methodically rigorous study design is necessary to elucidate the mechanisms regarding how these physiological variables lead to ABI in continuous blood flow under the ECMO circuit.

Early assessment and recognition of myocardial function using low PP during the first 24 hours of ECMO support has important clinical implications. Recognizing low PP may allow providers to promptly develop appropriate management techniques, including fine-tuning ECMO settings and using inotropes/vasopressors,^48^ LV venting,^49^ or pulsatile ECMO flow^50^ to improve hemodynamics. Notably, our analysis showed that the use of vasopressors before ECMO support was protective of ABI, supporting our speculation. Additionally, upon recognizing low PP, clinicians can consider more vigilant and standardized neuromonitoring strategies to ensure adequate cerebral perfusion and prevent occurrence/worsening of ABI, which is especially important in peripheral VA-ECMO due to the potential for differential oxygenation.^51^

### Limitations

Our analysis was retrospective and observational, thus limiting our ability to determine causation effects. Additionally, the ELSO Registry lacks granular ABG data, only allowing us to extract one pre-ECMO and one on-ECMO data point for each patient in our analysis. Similarly, for PP we are limited to a single value at 24 hours, however, it is unclear if there is significant variance over the first 24 hours that would influence interpretation. This methodology has also been previously validated in an ELSO Registry analysis of PP and mortality in 2,400 VA-ECMO patients.^52^ Furthermore, it is unclear how such variance would systematically bias our results as any variation should be at random and thus favor the null hypothesis. Additionally, low PP may represent a population with higher severity of illness which may explain the higher occurrence of ABI despite attempting to account for this with statistical modeling. The ELSO Registry also does not contain specific anticoagulation data, which is a known risk factor for ABI. Nevertheless, we adjusted for many ECMO-specific and clinically relevant covariates in our analysis, and low PP was still independently associated with ABI. Additionally, our study represents the largest and most comprehensive analysis to-date investigating the association between PP and ABI in VA-ECMO patients. We also used methodically rigorous methods in our analysis including multiple imputation to handle missing data, thus minimizing bias and invigorating the validity of our analysis^53^ and cubic spline analysis when identifying a PP threshold to abate the loss of information and poor predictions when using continuous variables.^54^ We also excluded ECMO patients simultaneously on LV venting devices as LV venting can directly modulate the PP in ECMO and thus could potentially confounding our findings.^3^ Finally, the optimum ECMO pump flow rate based on body surface area for each patient was not able to be determined in the ELSO Registry and should be noted.

## Conclusions

In the largest analysis to-date of peripheral VA-ECMO patients with CS, a PP reading of 10 mmHg or less at the 24-hour time point of ECMO support was associated with increased occurrence of ABI. Low early PP was also uniquely associated with CNS ischemia but not ICH. Accordingly, PP during ECMO support may serve as a distinct marker for ABI in this high-risk population. Given these findings, prospective observational studies investigating the association between PP and ABI with granular data and standardized neurological diagnoses is warranted.

## Figures and Tables

**Table 1.** Baseline characteristics and clinical variables of venoarterial extracorporeal membrane oxygenation patients with cardiogenic shock stratified by pulse pressure.

	Total (n=9,807)	Pulse Pressure > 10 mm Hg (n=8,294, 85%)	Pulse Pressure ≤ 10 mm Hg (1=1,513, 15%)	P-value
**Demographics**				
Age (years)	57.4 (45.9–65.7)	57.3 (45.7–65.7)	57.7 (46.5–65.4)	0.46
Male sex	6,661 (67%)	5,640 (68%)	1,021 (67%)	0.71
Body Mass Index, kg/m^2^	28.3 (24.6–33.0)	28.3 (24.5–33.1)	28.3 (24.9–32.4)	0.65
**Race/ethnicity**				0.36
Asian	1,099 (11%)	949 (11%)	150 (10%)	
Black	1,286 (13%)	1,071 (13%)	215 (14%)	
Hispanic	640 (7%)	543 (7%)	97 (6%)	
White	5,448 (56%)	4,605 (56%)	843 (56%)	
Others	1,334 (14%)	1,126 (14%)	208 (14%)	
**Year ECLS**				0.15
2018	1,360 (14%)	1,149 (14%)	211 (14%)	
2019	1,807 (18%)	1,559 (19%)	248 (16%)	
2020	1,780 (18%)	1,492 (18%)	288 (19%)	
2021	1,919 (20%)	1,633 (20%)	286 (19%)	
2022	2,166 (22%)	1,803 (22%)	363 (24%)	
2023	775 (8%)	658 (8%)	117 (8%)	
**Past medical history**				
Diabetes	1,161 (12%)	985 (12%)	176 (12%)	0.82
Hypertension	1,759 (18%)	1,498 (18%)	261 (17%)	0.47
Atrial fibrillation	1,211 (12%)	1,049 (13%)	162 (11%)	***0.04***
Cardiomyopathy	995 (10%)	855 (10%)	140 (9%)	0.23
COPD	281 (3%)	244 (3%)	37 (2%)	***<0.001***
COVID-19 status	242 (2%)	185 (2%)	57 (4%)	***<0.001***
**Pre-ECMO support**				
Additional temporary mechanical circulatory support	3,977 (41%)	3,262 (39%)	715 (47%)	***<0.001***
Vasopressor infusions	6,967 (71%)	5,885 (71%)	1,082 (72%)	0.68
Inotrope infusions	3,752 (38%)	3,222 (39%)	530 (35%)	***0.005***
**Pre-ECMO blood pressure variables**				
Systolic blood pressure (mm Hg)	91 (77–108)	92 (78–109)	86 (72–102)	***<0.001***
Diastolic blood pressure (mm Hg)	57 (47–68)	57 (46.3–68)	59 (48–71)	***<0.001***
Mean blood pressure (mm Hg)	69 (59–79)	69 (59–79)	67 (56.7–79)	***0.02***
Pulse pressure (mm Hg)	33 (22–45)	34 (23–47)	26 (16–38)	***<0.001***
Mean arterial pressure (mm Hg)	13 (10–16)	13 (10–16)	13 (10–17)	0.49
**Pre-ECMO ABG**				
pH	7.28 (7.18–7.37)	7.29 (7.18–7.37)	7.3 (7.2–7.4)	***0.001***
HCO_3_-(mEq/L)	19 (15–22.4)	19 (15.2–22.6)	18 (14–22)	***<0.001***
PaO_2_ (mm Hg)	103 (71–188)	102 (71–187)	107 (71–192)	0.23
PaCO_2_ (mm Hg)	40 (32–48.8)	40 (32.2–48.8)	39 (32–48.8)	0.13
Lactate (mmol/L)	6.3 (3.1–11)	6 (3–10.6)	7.8 (3.9–12)	***<0.001***
SpO_2_ (%)	97 (92–100)	97 (92–100)	97 (91–100)	0.41
SaO_2_ (%)	97 (92−99)	97 (92–99)	97 (92–99)	0.58
**On-ECMO blood pressure variables**				
Systolic blood pressure (mm Hg)	95 (83–108)	98 (88–111)	76 (69–83)	***<0.001***
Diastolic blood pressure (mm Hg)	64 (57–72)	63 (56–71)	70 (63–78)	***<0.001***
Mean blood pressure (mm Hg)	74 (67–81)	74 (68–82)	72 (65–80)	***<0.001***
Pulse pressure (mm Hg)	30 (17–44)	34 (23–47)	5 (3–8)	***<0.001***
Mean arterial pressure (mm Hg)	12 (10–14)	12 (10–14)	12 (10–15)	***<0.001***
**On-ECMO ABG**				
pH	7.42 (7.38–7.47)	7.43 (7.38–7.47)	7.41 (7.36–7.46)	***<0.001***
HCO_3_-(mEq/L)	24.1 (21.8–27)	24.3 (22–27)	24 (21–27)	***<0.001***
PaO_2_ (mm Hg)	132.8 (90–224)	125 (87.9–200)	202.5 (118–342.8)	***<0.001***
PaCO_2_ (mm Hg)	37.5 (33–42)	37.1 (33–42)	38 (34–42.35)	***<0.001***
Lactate (mmol/L)	2.1 (1.3–3.8)	2 (1.3–3.4)	2.8 (1.5–5.4)	***<0.001***
SpO_2_ (%)	99 (97–100)	99 (97–100)	99 (97–100)	***0.004***
SaO_2_ (%)	98 (97–99)	98 (97–99)	99 (98–100)	***<0.001***
**ΔPaCO_2_**	−2 (−11.3–6)	−2.1 (−11.43–5.8)	−1 (−11–7.05)	***0.009***
**Pump flow rate (4 hours, L/min)**	3.8 (3.2–4.37)	3.8 (3.2–4.34)	3.9 (3.2–4.45)	***0.007***
**Pump flow rate (24 hours, L/min)**	3.92 (3.28–4.47)	3.9 (3.24–4.43)	4.01 (3.43–4.54)	***<0.001***
**Days on ECMO support**	4.9 (2.9–8)	4.88 (2.96–6.48)	5.04 (2.58–8.79)	0.78
**Neurological complications on-ECMO**				
*Composite ABI*	1,096 (11%)	876 (11%)	220 (15%)	***<0.001***
*Composite Ischemia*	608 (6%)	487 (6%)	121 (8%)	***0.002***
*Ischemia*	207 (2%)	168 (2%)	39 (3%)	0.20
*Infarction*	412 (4%)	329 (4%)	83 (5%)	***0.008***
*Composite ICH*	311 (3%)	252 (3%)	59 (4%)	0.09
Intra/extra parenchymal hemorrhage	193 (2%)	155 (2%)	38 (3%)	0.12
Intraventricular hemorrhage	71 (1%)	62 (1%)	9 (1%)	0.63
Brain death	167 (2%)	124 (1%)	43 (3%)	***<0.001***
Neurosurgical intervention	24 (1%)	22 (1%)	2 (1%)	0.57
Seizures confirmed by EEG	110 (1%)	88 (1%)	22 (1%)	0.85
Seizures clinically determined	105 (1%)	90 (1%)	15 (1%)	0.23
**Other complications on-ECMO**				
ECMO circuit mechanical failure	994 (10%)	852 (10%)	142 (9%)	0.65
Renal replacement theory	3,495 (36%)	2,862 (35%)	633 (42%)	***<0.001***
Hemolysis	463 (5%)	378 (5%)	85 (6%)	0.085
Cardiac arrhythmia	1,479 (15%)		283 (19%)	***<0.001***
Gastrointestinal hemorrhage	527 (5%)	415 (5%)	112 (7%)	***<0.001***
**Outcomes**				
In-hospital mortality	4,834 (49%)	3,813 (46%)	1,021 (67%)	***<0.001***

Δ = delta

**Table 2.** Risk factors associated with *acute brain injury* in multivariable logistic regression analysis in peripheral VA-ECMO patients with cardiogenic shock.

	aOR	Lower 95% CI	Upper 95% CI	*P*-value
**Age (by 10 years)**	1.04	0.99	1.08	0.16
**Female sex**	0.92	0.80	1.05	0.22
**Body mass index (by 10 kg/m^2^)**	1.15	0.52	2.53	0.72
**Pre-ECMO variables**	1.04	0.98	1.11	0.23
Additional temporary mechanical circulatory support	0.99	0.86	1.13	0.84
Vasopressor infusions	0.90	0.78	1.03	0.13
Cardiac arrest	2.05	1.78	2.36	**<0.001**
**On-ECMO variables**				
**Pulse pressure ≤ 10 mm Hg**	1.25	1.06	1.48	**0.01**
PaO_2_ (by 10 mm Hg)	0.991	0.986	0.996	**<0.001**
pH (decreasing, per 0.1 units)	7.59	0.002	2.25E4	0.62
**Delta PaCO_2_ (by 10 mm Hg)**	1.10	1.05	1.15	**<0.001**
**On-ECMO Complications**				
Hemolysis	1.81	1.42	2.32	**<0.001**
Arrhythmia	1.41	1.19	1.66	**<0.001**
Renal replacement therapy	1.41	1.23	1.61	**<0.001**

Missing values were handled with multiple imputations to increase statistical power. Pre-ECMO temporary mechanical circulatory support consisted of an intra-aortic balloon pump, Impella, and left ventricular assist devices. Pre-ECMO vasopressor infusions included dopamine, epinephrine, norepinephrine, phenylephrine, and vasopressin. Hemolysis was defined as a peak plasma hemoglobin of at least 50 mg/dL occurring at least once during the ECMO run and sustained for at least 2 consecutive days. aOR: adjusted odds ratio CI: confidence interval. ECMO: extracorporeal membrane oxygenation. PaO_2_: arterial partial pressure of oxygen. PaCO_2_: arterial partial pressure of carbon dioxide.

## Abbreviations

ABG: arterial blood gas
ABI: acute brain injury
aOR: adjusted odds ratio
CI: confidence interval
CNS: central nervous system
CS: cardiogenic shock
DBP: diastolic blood pressure
ECMO: extracorporeal membrane oxygenation
ELSO: Extracorporeal Life Support Organization
IABP: intra-aortic balloon pump
ICH: intracranial hemorrhage
IQR: interquartile range
LV: left ventricular
LVAD: left ventricular assist device
PaO_2_: partial pressure of oxygen
PP: pulse pressure
SD: standard deviation
SBP: systolic blood pressure
tMCS: temporary mechanical circulatory support

## References

[1] Jentzer JC, Baran DA, Kyle Bohman J, Cardiogenic shock severity and mortality in patients receiving venoarterial extracorporeal membrane oxygenator support. Eur Heart J Acute Cardiovasc Care. 2022;11:891–903. 36173885 10.1093/ehjacc/zuac119 PMC13376136

[2] Jentzer JC, Miller PE, Alviar C, Yalamuri S, Bohman JK, Tonna JE. Exposure to Arterial Hyperoxia During Extracorporeal Membrane Oxygenator Support and Mortality in Patients With Cardiogenic Shock. Circ Heart Fail. 2023;16:e010328. 36871240 10.1161/CIRCHEARTFAILURE.122.010328 PMC10121893

[3] Thiagarajan RR, Barbaro RP, Rycus PT, Extracorporeal Life Support Organization Registry International Report 2016. ASAIO J. 2017;63:60–67. 27984321 10.1097/MAT.0000000000000475

[4] Eckman PM, Katz JN, El Banayosy A, Bohula EA, Sun B, van Diepen S. Veno-Arterial Extracorporeal Membrane Oxygenation for Cardiogenic Shock: An Introduction for the Busy Clinician. Circulation. 2019;140:2019–2037. 31815538 10.1161/CIRCULATIONAHA.119.034512

[5] Cho SM, Canner J, Chiarini G, Modifiable Risk Factors and Mortality From Ischemic and Hemorrhagic Strokes in Patients Receiving Venoarterial Extracorporeal Membrane Oxygenation: Results From the Extracorporeal Life Support Organization Registry. Crit Care Med. 2020;48:e897–e905. 32931195 10.1097/CCM.0000000000004498 PMC8170265

[6] Homan TD, Bordes SJ, Cichowski E. Physiology, Pulse Pressure. StatPearls. Treasure Island (FL): StatPearls Publishing Copyright © 2023, StatPearls Publishing LLC.; 2023. 29494015

[7] Rilinger J, Riefler AM, Bemtgen X, Impact of pulse pressure on clinical outcome in extracorporeal cardiopulmonary resuscitation (eCPR) patients. Clin Res Cardiol. 2021;110:1473–1483. 33779810 10.1007/s00392-021-01838-7 PMC8405467

[8] Lee SI, Lim YS, Park CH, Choi WS, Choi CH. Importance of pulse pressure after extracorporeal cardiopulmonary resuscitation. J Card Surg. 2021;36:2743–2750. 33993537 10.1111/jocs.15614

[9] O’Neil MP, Fleming JC, Badhwar A, Guo LR. Pulsatile versus nonpulsatile flow during cardiopulmonary bypass: microcirculatory and systemic effects. Ann Thorac Surg. 2012;94:2046–2053. 22835552 10.1016/j.athoracsur.2012.05.065

[10] Purohit SN, Cornwell WK 3rd, Pal JD, Lindenfeld J, Ambardekar AV. Living Without a Pulse: The Vascular Implications of Continuous-Flow Left Ventricular Assist Devices. Circ Heart Fail. 2018;11:e004670. 29903893 10.1161/CIRCHEARTFAILURE.117.004670 PMC6007027

[11] Shou BL, Wilcox C, Florissi I, Early Low Pulse Pressure in VA-ECMO Is Associated with Acute Brain Injury. Neurocrit Care. 2022. 10.1007/s12028-022-01607-y PMC10040467 36167950

[12] Wilcox C, Etchill E, Giuliano K, Acute Brain Injury in Postcardiotomy Shock Treated With Venoarterial Extracorporeal Membrane Oxygenation. Journal of Cardiothoracic and Vascular Anesthesia. 2021;35:1989–1996. 33593649 10.1053/j.jvca.2021.01.037

[13] Gu K, Zhang Y, Gao B, Chang Y, Zeng Y. Hemodynamic Differences Between Central ECMO and Peripheral ECMO: A Primary CFD Study. Med Sci Monit. 2016;22:717–726. 26938949 10.12659/MSM.895831 PMC4780269

[14] Lorusso R, Alexander P, Rycus P, Barbaro R. The Extracorporeal Life Support Organization Registry: update and perspectives. Ann Cardiothorac Surg. 2019;8:93–98. 30854317 10.21037/acs.2018.11.03 PMC6379190

[15] ELSO. Extracorporeal Life Support Organization (ELSO) Registry Data Definitions2018.

[16] Rubin DB. Inference and missing data. Biometrika. 1976;63:581–592.

[17] Shou BL, Ong CS, Premraj L, Arterial oxygen and carbon dioxide tension and acute brain injury in extracorporeal cardiopulmonary resuscitation patients: Analysis of the extracorporeal life support organization registry. J Heart Lung Transplant. 2023;42:503–511. 36435686 10.1016/j.healun.2022.10.019 PMC10050131

[18] Su Y, Liu K, Zheng J-L, Hemodynamic monitoring in patients with venoarterial extracorporeal membrane oxygenation. Annals of Translational Medicine. 2020;8:792. 32647717 10.21037/atm.2020.03.186 PMC7333156

[19] Pinsino A, Mondellini GM, Castagna F, Estimation of Mean Arterial Pressure Using Doppler and Pump Parameters in HeartMate 3 Patients. The Journal of Heart and Lung Transplantation. 2020;39:S156.

[20] Estep JD, Trachtenberg BH, Loza LP, Bruckner BA. Continuous flow left ventricular assist devices: shared care goals of monitoring and treating patients. Methodist Debakey Cardiovasc J. 2015;11:33–44. 25793028 10.14797/mdcj-11-1-33 PMC4362063

[21] Wilcox C, Choi CW, Cho S-M. Brain injury in extracorporeal cardiopulmonary resuscitation: translational to clinical research. Journal of Neurocritical Care. 2021;14:63–77.

[22] Crow S, John R, Boyle A, Gastrointestinal bleeding rates in recipients of nonpulsatile and pulsatile left ventricular assist devices. J Thorac Cardiovasc Surg. 2009;137:208–215. 19154927 10.1016/j.jtcvs.2008.07.032

[23] Veraar CM, Rinosl H, Kuhn K, Non-pulsatile blood flow is associated with enhanced cerebrovascular carbon dioxide reactivity and an attenuated relationship between cerebral blood flow and regional brain oxygenation. Crit Care. 2019;23:426. 31888721 10.1186/s13054-019-2671-7 PMC6937980

[24] Stöhr EJ, McDonnell BJ, Colombo PC, Willey JZ. CrossTalk proposal: Blood flow pulsatility in left ventricular assist device patients is essential to maintain normal brain physiology. J Physiol. 2019;597:353–356. 30560570 10.1113/JP276729 PMC6332785

[25] Wadowski PP, Steinlechner B, Zimpfer D, Functional capillary impairment in patients with ventricular assist devices. Sci Rep. 2019;9:5909. 30976042 10.1038/s41598-019-42334-3 PMC6459831

[26] Roach GW, Kanchuger M, Mangano CM, Adverse cerebral outcomes after coronary bypass surgery. Multicenter Study of Perioperative Ischemia Research Group and the Ischemia Research and Education Foundation Investigators. N Engl J Med. 1996;335:1857–1863. 8948560 10.1056/NEJM199612193352501

[27] Acharya D, Loyaga-Rendon R, Morgan CJ, INTERMACS Analysis of Stroke During Support With Continuous-Flow Left Ventricular Assist Devices: Risk Factors and Outcomes. JACC Heart Fail. 2017;5:703–711. 28958345 10.1016/j.jchf.2017.06.014 PMC5743224

[28] Fendler TJ, Spertus JA, Gosch KL, Incidence and predictors of cognitive decline in patients with left ventricular assist devices. Circ Cardiovasc Qual Outcomes. 2015;8:285–291. 25925372 10.1161/CIRCOUTCOMES.115.001856 PMC4418227

[29] Caro MA, Rosenthal JL, Kendall K, Pozuelo L, Funk MC. What the Psychiatrist Needs to Know About Ventricular Assist Devices: A Comprehensive Review. Psychosomatics. 2016;57:229–237. 27005723 10.1016/j.psym.2016.01.002

[30] Serraino GF, Marsico R, Musolino G, Pulsatile cardiopulmonary bypass with intra-aortic balloon pump improves organ function and reduces endothelial activation. Circ J. 2012;76:1121–1129. 22447003 10.1253/circj.cj-11-1027

[31] Hilbert T, Duerr GD, Hamiko M, Endothelial permeability following coronary artery bypass grafting: an observational study on the possible role of angiopoietin imbalance. Crit Care. 2016;20:51. 26951111 10.1186/s13054-016-1238-0 PMC4782352

[32] Ellermann SF TW LS, Jongman RM, Plasma from patients undergoing coronary artery bypass graft surgery does not activate endothelial cells under shear stress in vitro. Int J Crit Illn Inj Sci. 2021;11:142–150. 34760660 10.4103/IJCIIS.IJCIIS_197_20 PMC8547679

[33] Onorati F, Rubino AS, Nucera S, Off-pump coronary artery bypass surgery versus standard linear or pulsatile cardiopulmonary bypass: endothelial activation and inflammatory response. European Journal of Cardio-Thoracic Surgery. 2010;37:897–904. 20018523 10.1016/j.ejcts.2009.11.010

[34] Lanzarone E, Gelmini F, Tessari M, Preservation of endothelium nitric oxide release by pulsatile flow cardiopulmonary bypass when compared with continuous flow. Artif Organs. 2009;33:926–934. 19860792 10.1111/j.1525-1594.2009.00888.x

[35] Hutcheson IR, Griffith TM. Release of endothelium-derived relaxing factor is modulated both by frequency and amplitude of pulsatile flow. Am J Physiol. 1991;261:H257–262. 1858928 10.1152/ajpheart.1991.261.1.H257

[36] Boyle EM Jr,., Pohlman TH, Johnson MC, Verrier. Endothelial cell injury in cardiovascular surgery: the systemic inflammatory response. Ann Thorac Surg. 1997;63:277–284. 8993292 10.1016/s0003-4975(96)01061-2

[37] O’Neil MP, Alie R, Guo LR, Myers ML, Murkin JM, Ellis CG. Microvascular Responsiveness to Pulsatile and Nonpulsatile Flow During Cardiopulmonary Bypass. Ann Thorac Surg. 2018;105:1745–1753. 29391150 10.1016/j.athoracsur.2018.01.007

[38] Shou BL, Ong CS, Zhou AL, Arterial Carbon Dioxide and Acute Brain Injury in Venoarterial Extracorporeal Membrane Oxygenation. ASAIO J. 2022;68:1501–1507. 35671442 10.1097/MAT.0000000000001699 PMC9477972

[39] Al-Kawaz MN, Canner J, Caturegli G, Duration of Hyperoxia and Neurologic Outcomes in Patients Undergoing Extracorporeal Membrane Oxygenation. Crit Care Med. 2021;49:e968–e977. 33935164 10.1097/CCM.0000000000005069

[40] Al-Kawaz M, Shou B, Prokupets R, Whitman G, Geocadin R, Cho SM. Mild hypothermia and neurologic outcomes in patients undergoing venoarterial extracorporeal membrane oxygenation. J Card Surg. 2022;37:825–830. 35152478 10.1111/jocs.16308 PMC8891050

[41] Inamori S, Shirai M, Yahagi N, A comparative study of cerebral microcirculation during pulsatile and nonpulsatile selective cerebral perfusion: assessment by synchrotron radiation microangiography. ASAIO J. 2013;59:374–379. 23820275 10.1097/MAT.0b013e3182976939

[42] Le Guennec L, Cholet C, Huang F, Ischemic and hemorrhagic brain injury during venoarterial-extracorporeal membrane oxygenation. Ann Intensive Care. 2018;8:129. 30570687 10.1186/s13613-018-0475-6 PMC6301905

[43] Illum B, Odish M, Minokadeh A, Evaluation, Treatment, and Impact of Neurologic Injury in Adult Patients on Extracorporeal Membrane Oxygenation: a Review. Current Treatment Options in Neurology. 2021;23:15. 33814895 10.1007/s11940-021-00671-7 PMC8009934

[44] Kalra A, Shou BL, Zhao D, Racial and ethnical discrepancy in hypoxemia detection in patients on extracorporeal membrane oxygenation. JTCVS Open. 10.1016/j.xjon.2023.02.011 PMC10328809 37425474

[45] Kalra A, Shou BL, Zhao D, ECMO Physiological Factors Influence Pulse Oximetry and Arterial Oxygen Saturation Discrepancies. The Annals of Thoracic Surgery. 10.1016/j.athoracsur.2023.09.019 PMC10959762 37748529

[46] Asher SR, Curry P, Sharma D, Survival advantage and PaO_2_ threshold in severe traumatic brain injury. J Neurosurg Anesthesiol. 2013;25:168–173. 23343758 10.1097/ANA.0b013e318283d350

[47] Altman DG, Royston P. The cost of dichotomising continuous variables. Bmj. 2006;332:1080. 16675816 10.1136/bmj.332.7549.1080 PMC1458573

[48] Harper MD, Maybauer MO. Vasopressor and Inotropic Support in ECMO Patients With Refractory Shock. Extracorporeal Membrane Oxygenation: An Interdisciplinary Problem-Based Learning Approach: Oxford University Press; 2022:0.

[49] Cevasco M, Takayama H, Ando M, Garan AR, Naka Y, Takeda K. Left ventricular distension and venting strategies for patients on venoarterial extracorporeal membrane oxygenation. J Thorac Dis. 2019;11:1676–1683. 31179113 10.21037/jtd.2019.03.29 PMC6531683

[50] Kanagarajan D, Heinsar S, Gandini L, Preclinical Studies on Pulsatile Veno-Arterial Extracorporeal Membrane Oxygenation: A Systematic Review. ASAIO Journal. 2023;69:e167–e180. 36976324 10.1097/MAT.0000000000001922

[51] Cove ME. Disrupting differential hypoxia in peripheral veno-arterial extracorporeal membrane oxygenation. Crit. Care. 2015;19:280. 27391473 10.1186/s13054-015-0997-3 PMC4511033

[52] Rali AS, Ranka S, Butcher A, Early Blood Pressure Variables Associated With Improved Outcomes in VA-ECLS: The ELSO Registry Analysis. JACC Heart Fail. 2022;10:397–403. 35654524 10.1016/j.jchf.2022.04.003 PMC9214574

[53] Sterne JA, White IR, Carlin JB, Multiple imputation for missing data in epidemiological and clinical research: potential and pitfalls. Bmj. 2009;338:b2393. 19564179 10.1136/bmj.b2393 PMC2714692

[54] Gauthier J, Wu QV, Gooley TA. Cubic splines to model relationships between continuous variables and outcomes: a guide for clinicians. Bone Marrow Transplant. 2020;55:675–680. 31576022 10.1038/s41409-019-0679-x

